# Mitochondrial and energy metabolism dysfunctions are hallmarks of TDP-43^G376D^ fibroblasts from members of an Amyotrophic Lateral Sclerosis family

**DOI:** 10.1038/s41419-025-07584-2

**Published:** 2025-04-10

**Authors:** Elisa Perciballi, Federica Bovio, Sara Ferro, Matilde Forcella, Jessica Rosati, Rose Mary Carletti, Angela D’Anzi, Maurizio Gelati, Vincenzo La Bella, Metello Innocenti, Rossella Spataro, Martina Pecoraro, Ivan Lombardi, Edvige Vulcano, Giorgia Ruotolo, Sara Mercurio, Mario Sabatelli, Serena Lattante, Tarja Malm, Sohvi Ohtonen, Angelo Luigi Vescovi, Paola Fusi, Daniela Ferrari

**Affiliations:** 1 Institute for Stem-Cell Biology, Regenerative Medicine and Innovative Therapies (ISBReMIT), Production Unit of Advanced Therapies (UPTA), Fondazione IRCCS Casa Sollievo della Sofferenza, Viale dei Cappuccini 1, 71013 San Giovanni Rotondo, Italy; 2 Department of Biotechnology and Biosciences, University of Milano-Bicocca, P.zza della Scienza, 2, 20126 Milan, Italy; 3 Cellular Reprogramming Unit, Fondazione IRCCS Casa Sollievo della Sofferenza, Viale dei Cappuccini 1, 71013 San Giovanni Rotondo, Italy; 4 Saint Camillus International, University of Health Sciences, Rome, Italy; 5 ALS Clinical Research Centre and Laboratory of Neurochemistry, Department of Experimental Biomedicine and Clinical Neurosciences, University of Palermo, Via del Vespro, 129, 90127 Palermo, Italy; 6 Intensive Neurorehabilitation Unit, Villa delle Ginestre Hospital, Via Castellana, 145 – 90135 Palermo, Italy; 7 Adult NEMO Clinical Center, Fondazione Policlinico Universitario A. Gemelli IRCCS, Largo A. Gemelli 8, 00168 Rome, Italy; 8 Section of Neurology, Università Cattolica del Sacro Cuore, Largo Francesco Vito, 1, 00168 Rome, Italy; 9 Unit of Medical Genetics, Università Cattolica del Sacro Cuore, Fondazione Policlinico Universitario A. Gemelli IRCCS, Largo A. Gemelli 8, 00168 Rome, Italy; 10 A.I. Virtanen Institute for Molecular Sciences, University of Eastern Finland, Neulaniementie, 2, FI-70211 Kuopio, Finland; 11 Abu Dhabi Stem Cells Center, Abu Dhabi, United Arab Emirates

**Keywords:** Amyotrophic lateral sclerosis, Neurochemistry, Disease genetics, Mechanisms of disease

## Abstract

Amyotrophic Lateral Sclerosis (ALS) is an incurable neurodegenerative disease, causing degeneration of motor neurons, paralysis, and death. About 5–10% of cases are associated with gene mutations inherited from a family member (fALS). Among them, mutations in the transactive-response (TAR)-DNA-binding protein (*TARDBP*), which encodes for the TAR DNA binding protein 43 (TDP-43) are responsible for 4–5% of fALS but the molecular mechanisms that initiate and sustain the neurodegenerative process are largely unknown. Metabolic impairments might be involved in the pathogenesis of ALS and are currently under investigation. In order to correlate biochemical and metabolic alterations with disease progression, here, we established the metabolic fingerprint of dermal fibroblasts derived from symptomatic and asymptomatic members of a family with fALS cases carrying to the p.G376D mutation in TDP-43. We found that increased proliferation, unbalanced oxidative homeostasis and higher ATP production rate coupled with enhanced metabolic activity are underlying traits of this family. Fibroblasts from carrier individuals deploy several mechanisms to increase mitochondrial respiration to meet increasing energy demands. This is accompanied by an upregulation of glycolysis corresponding to a metabolic reprograming towards a glycolytic phenotype for ATP production during ALS progression, particularly in late disease stages. In summary, we uncover alterations in energy metabolism in TDP43^G376D^ patient-derived primary fibroblasts that may be used as risk biomarkers and/or to monitor ALS progression.

## Introduction

Amyotrophic Lateral Sclerosis (ALS) is a fatal neurodegenerative disease, whose clinical course is mostly driven by the selective degeneration of both upper and lower motor neurons (MNs), at the level of motor cortex, brainstem and spinal cord. Patients suffer from a progressive generalized paralysis [1] that leads to death predominantly in 3–5 years from diagnosis [2]. ALS presents both sporadic (sALS, 90%) and familial (fALS, 5-10%) forms [3]. Several mutated genes are known risk factors, among which *SOD1, TARDBP, FUS, ANG, OPTN* and C9orf72 are the most frequent [4].

The causes of disease onset and progression are still unknown, and diverse possible mechanisms [5] are under investigation, including increased oxidative stress [6] and mitochondrial damage [7, 8]. Moreover, oxidative stress might affect mitochondria function/activity [9] and alter energy metabolism. Indeed, energetic imbalance is being investigated as a potential element of disease pathogenesis in patients [10] and mouse models of ALS, both at the systemic [11] and cellular levels [12–15].

ALS patients display heterogenous phenotypes and ALS-causative mutations have a highly variable penetrance in fALS families. This is the case of the c.1127 G→A mutation in the *TARDBP* gene, identified in three families [16, 17], which include both symptomatic and asymptomatic carriers. This mutation results in a glycine-to-aspartate substitution (p.Gly376Asp) in the C-terminal domain [18] of the TAR DNA binding protein 43 (TDP-43^G376D^) and is associated with a fast disease progression and a dominant pattern of transmission with incomplete penetrance.

TDP-43 is a nuclear RNA/DNA binding protein that can shuttle between the nucleus and the cytoplasm to exert its regulatory role in RNA-related metabolism [19]. Most mutations (including the p.Gly376Asp) promote cytoplasmic mislocalization [20] and formation of toxic protein aggregates [21], coupled to a depletion TDP-43 in the nucleus that causes a loss of the nuclear function of the protein [22]. However, the mechanisms by which mutated TDP-43 causes a pathogenetic effect are still to be elucidated. It has recently been proposed that TDP-43 aggregation might have a role in the impairment of energy metabolism and cellular respiration. Indeed, the activity of the complexes involved in the electron transport chain have been shown to be decreased upon mutation of *TARDBP* and other ALS-related genes [23].

Given this, we decided to study the possible interaction between genetic background and metabolic imbalance and its correlation with disease features such as susceptibility, age of onset, and progression rate/survival [24], capitalizing on primary dermal fibroblasts derived from the Italian *TARDBP* family [16]. The mean age of onset in this family is 50 years (IQR 37-57) and, even though the mutation is highly penetrant, more than 30% of the carriers are still asymptomatic at 70 years [25].

We compared oxidative stress vs antioxidant defenses, and energy metabolism in fibroblasts from a TDP-43^G376D^ patient, an asymptomatic TDP-43^G376D^ carrier, and both kin and non-kin healthy individuals. In addition to the identification of druggable disease mechanisms, better ways to stratify ALS patients and identify disease progression markers are urgently needed to better define specific disease stages and/or response to therapy. Therefore, we also analyzed fibroblasts derived from the same TDP-43^G376D^ fALS patient at early and late stages of the disease to gain information as to whether and how energy metabolism evolves during disease progression.

## Results

### Fibroblasts from a *TARDBP*-ALS family show an increased proliferation rate and a higher ATP production with respect to control cells

We collected a representative cohort of fibroblasts derived from an Italian fALS-family [16] with carriers of the c.1127 G→A mutation in the *TARDBP* gene (coding for TDP-43^G376D^) to study the role of *TARDBP* mutations in ALS and the contribution of familiar genetic traits to the disease. We obtained fibroblasts from: (i) a 34 years-old non-carrier female donor (Familial CTRL), (ii) a 34 years-old TDP-43^G376D^ carrier female donor who was asymptomatic at the time of biopsy and is still asymptomatic 5 years later. (TDP-43^G376D^ Asymptomatic), (iii) a male TDP-43^G376D^ ALS patient who was diagnosed at the age of 36 and died 7 years later (TDP-43^G376D^ Early-stage patient, and TDP-43^G376D^ Late-stage patient). ALS onset was characterized by shoulder girdle, neck muscles deficits, and a rapid bulbar evolution. Over the years, the patient experienced progressive spastic quadriplegia and anarthria up to the locked-in syndrome 5 years after the diagnosis and complete locked-in syndrome the year after. He also needed enteral nutrition through a gastrostomy and started mechanical invasive ventilation by tracheostomy within 12 months of the diagnosis. Skin biopsies were taken at two disease stages: 2 and 6 years after disease onset (at 38 and 42 years of age, respectively).

Fibroblasts from two non-kin healthy female volunteers (30 and 53 years-old) were used as external unrelated controls (Unrelated CTRLs).

**Fig. 1:**
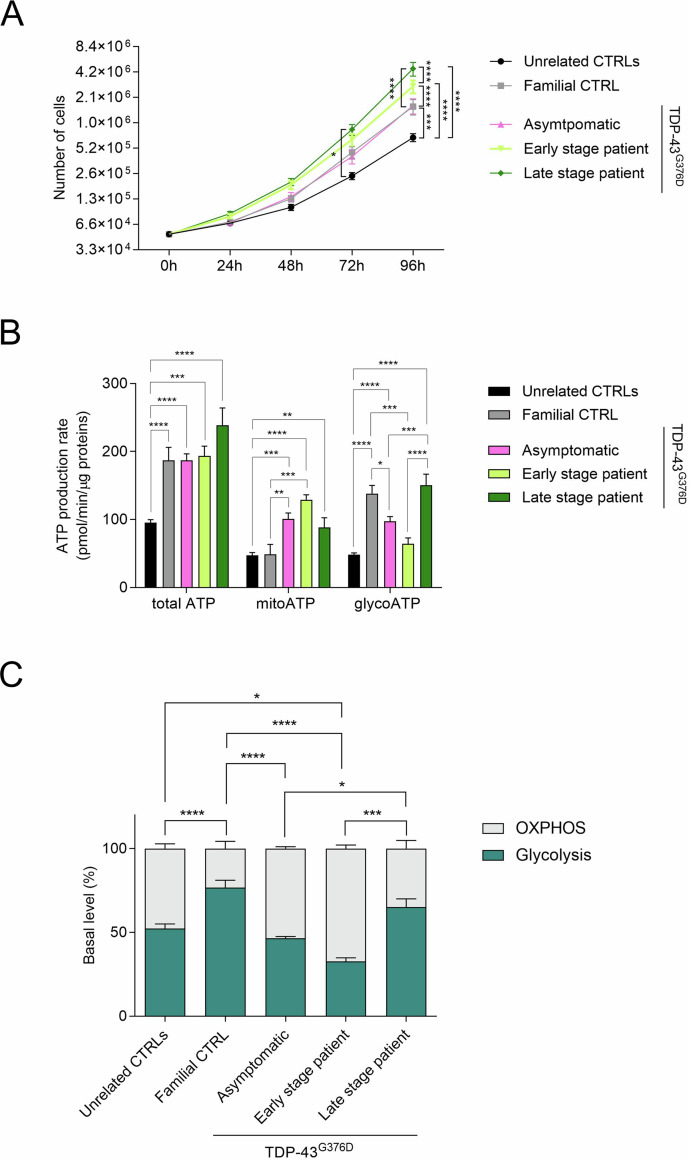
Evaluation of proliferation rate and ATP production in *TARDBP*-family fibroblasts. **A** Growth curves of TDP-43^G376D^ mutants and control fibroblasts. Data are shown as the mean ± SEM of four independent experiments. **B** ATP production rate expressed as pmol/min/µg protein in controls and TDP-43^G376D^ mutant fibroblasts, by means of Seahorse XF ATP Rate Assay Kit, in which 1.5 µM oligomycin A and 1 µM Rotenone together with 1 µM Antimycin A were provided in sequence. Data are shown as the mean ± SEM of at least three independent experiments. **C** Basal percentage level of glycolysis and oxidative phosphorylation. Statistical significance: *p < 0.05, **p < 0.01, ***p < 0.001 (Two-way ANOVA for (**A**) or One-way ANOVA test with Tukey’s correction).

By comparing the growth rates of all these cells, we observed that not only the fibroblasts derived from the *TARDBP* family displayed an increased growth rate compared to the unrelated controls, but also that ALS fibroblasts showed the faster growth, which increased from the early to the late disease stage (Fig. 1A). Given this evidence, we exploited Seahorse technology to measure ATP production rate. Although this technology does not allow a direct measure of the absolute amount of ATP and/or ADP, it has the advantage of conducting the analysis on live cells in real time and to assess the relative contribution to ATP production by glycolysis and oxidative phosphorylation. In accordance with these findings, fibroblasts from the *TARDBP* family had a significantly higher total ATP production rate than the unrelated controls (Fig. 1B). However, while the cells from the familial control donor relied primarily on glycolysis for ATP production, the TDP-43^G376D^ Asymptomatic fibroblasts used glycolysis and OXPHOS equally. Regarding patient’s fibroblasts, at the early-stage ATP is produced mainly through mitochondrial respiration, while at the late-stage ATP production relies more on glycolysis (Fig. 1B-C).

**Fig. 2:**
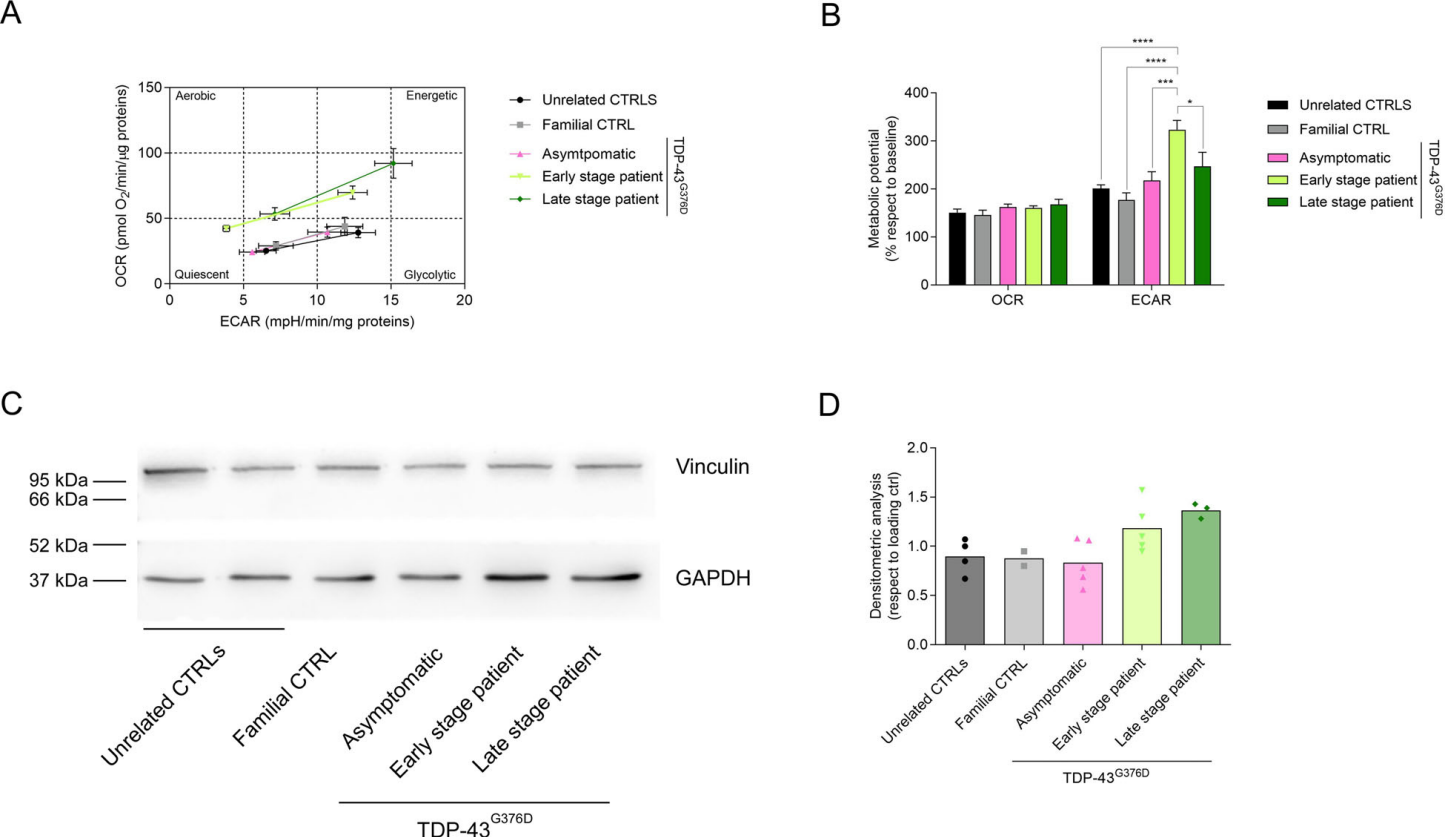
Cell energy phenotype evaluation via measurement of mitochondrial respiration (oxygen consumption rate, OCR) and glycolytic flux (extracellular acidification rate, ECAR) of ALS fibroblasts with those derived from healthy individuals or from asymptomatic carriers, both at baseline and upon exposure to stressors (by simultaneous administration of 1 µM oligomycin A and 2 µM FCCP). Energetic panel (**A**) and (**B**) metabolic potential of controls and TDP-43^G376D^ mutants from baseline conditions to stressed ones. Bars indicate the mean ± SEM of at least three independent experiments. Statistical significance: *p < 0.05, **p < 0.01 and ***p < 0.001 (One-way ANOVA test with Tukey’s correction). Western blot analysis of GAPDH (37 kDa) (**C**) and its expression levels in unrelated controls and TARDBP-ALS family fibroblasts expressed compared to loading control (Vinculin 121 kDa) (**D**).

Seahorse analysis of cell energetic profiles revealed that patient’s fibroblasts attained more energetic and more glycolytic phenotypes (Fig. 2A) with respect to all other cells. Moreover, under stress conditions, they could increase their glycolytic metabolic potential more than fibroblasts from other donors (Fig. 2B), probably due to a slight increase in the GAPDH protein level (Fig. 2C-D and Supplemental Material).

### TDP-43^G376D^ mutation enhances mitochondrial respiration

**Fig. 3:**
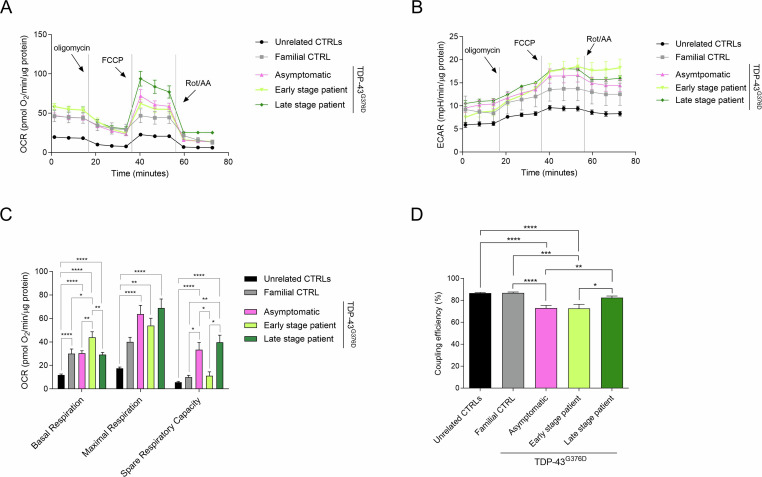
Evaluation of Mitochondrial functionality through Seahorse Mito Stress Test. OCR (**A**) and ECAR (**B**) profiles, expressed as pmol O2/min/µg proteins and mpH/min/µg proteins in control and TDP-43^G376D^ mutant fibroblasts, following the sequential addition of1 µM oligomycin A, 2 µM FCCP and 1 µM rotenone/antimycin A. Analysis of key mitochondrial parameters (**C**) and coupling efficiency (**D**). Bars indicate the mean ± SEM of at least three independent experiments replicates. Statistical significance: *p < 0.05, **p < 0.01, *** *p* < 0.001 (One-way ANOVA test with Tukey’s correction).

Given that metabolic dysfunctions could contribute to ALS pathogenesis and that mitochondria take center stage in cell metabolism, we further investigated mitochondrial bioenergetics through the Seahorse Mito Stress Test. The oxygen consumption rate (OCR) and extracellular acidification rate (ECAR) profiles revealed a higher oxygen consumption and extracellular acidification rate in the *TARDBP* family, compared to non-related control fibroblasts (Fig. 3A-B). However, although all *TARDBP* family fibroblasts showed a significantly enhanced basal and maximum respiration, compared to unrelated controls, they showed a peculiar mitochondrial respiration dynamic, which is also different between early and late-stage patients’ cells (Fig. 3C).

TDP-43^G376D^ Asymptomatic and Familial CTRL fibroblasts showed a similar basal respiration level; however, the former displayed a higher maximal respiration, leading to a statistically significant increase in the spare respiratory capacity (Fig. 3C). This suggests that these fibroblasts are not operating close to their bioenergetic limit and have a reserve of mitochondrial functionality. The early-stage patient fibroblasts showed a significantly higher basal respiration than TDP-43^G376D^ Asymptomatic fibroblasts, but a much lower spare respiratory capacity, whereas late-stage patient fibroblasts behaved as the TDP-43^G376D^ Asymptomatic fibroblasts, having similar basal respiration, maximal respiration, and spare respiratory capacity (Fig. 3C).

Intriguingly, TDP-43^G376D^ Asymptomatic and early-stage patient mitochondria were found to be less coupled than all the others, indicating the presence of mitochondrial stress, that could be counteracted in the early-to late-stage transition via glycolysis upregulation (Fig. 3D).

### *TARDBP*-family fibroblasts display an elevated oxidative stress burden respect to unrelated control fibroblasts

Increased oxidative stress is one of the main pathological features of ALS, including *TARDBP* fALS cases, and a possible driver of disease progression [26].

**Fig. 4:**
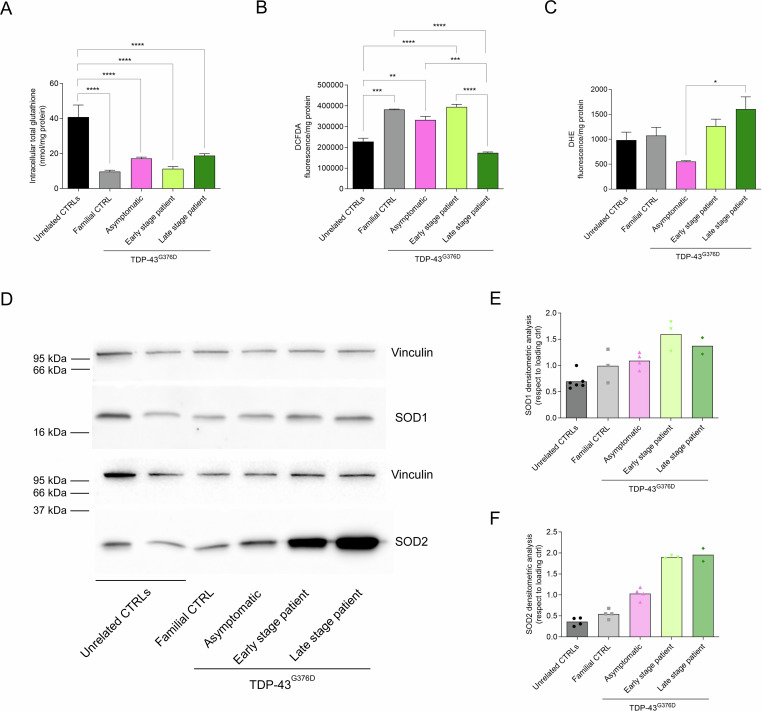
Analysis of oxidative stress. Total intracellular glutathione (**A**), total ROS (**B**), and cytosolic superoxide anion (O2^-^) content (**C**). Data represented the mean of at least three independent experiments and results are shown as the mean ± SEM. Statistical significance: *p < 0.05, **p < 0.01, ***p < 0.001, ****p < 0.0001 (One-way ANOVA test with Tukey’s correction). Representative Western blot (**D**) and the expression level of SOD1 (16 kDa) (**E**) and SOD2 (22 kDa) (**F**) and their expression levels in unrelated controls and TARDBP-ALS family fibroblasts compared to loading control (Vinculin 121 kDa).

Total glutathione, the main non-enzymatic antioxidant scavenger participating in cellular redox reactions [27], was found significantly higher in unrelated control cells than in all *TARDBP*-family fibroblasts, suggesting that a less efficient antioxidant system could be a family-related predisposing factor (Fig. 4A). Compared to the unrelated healthy controls fibroblasts, intracellular reactive oxygen species (ROS) were increased in all members of the *TARDBP* family, except the late-stage patient fibroblasts (Fig. 4B). Interestingly, late-stage patient fibroblasts showed a lower total ROS level than the early-stage ones, accompanied by increased cytosolic superoxide anion (O_2_^-^) contents (Fig. 4B, C). The increase in O_2_^-^ level was mirrored by the expression levels of superoxide dismutases (Fig. 4D–F and Supplemental Material), in particular the mitochondrial SOD2 was enhanced in patient fibroblasts, both at early and late disease stages.

### Morphometric analysis of mitochondria in ALS fibroblasts

**Fig. 5:**
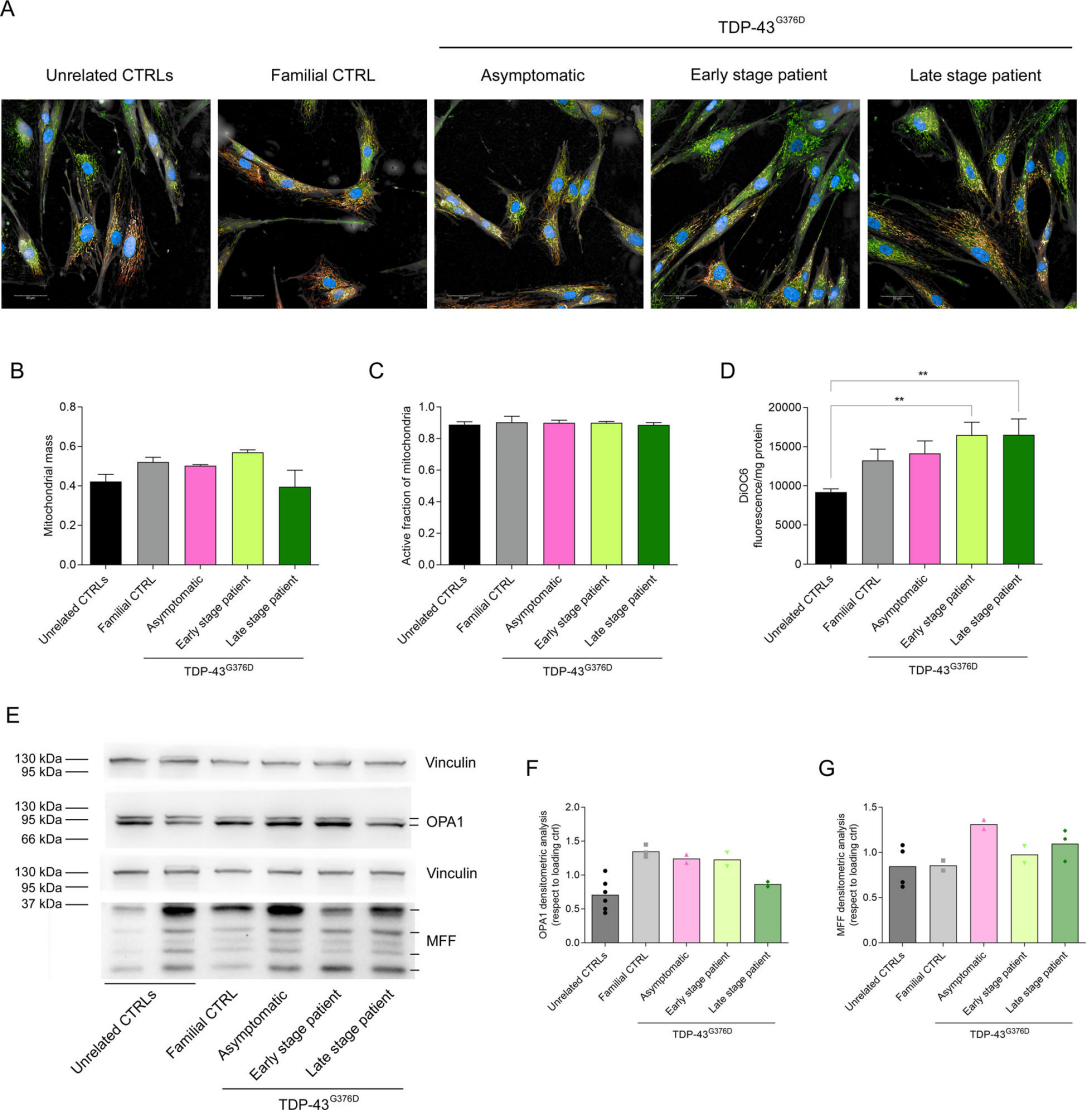
Morphometric analysis of mitochondria in *TARDBP*-ALS fibroblasts. Mitochondrial network in TARDBP-ALS family (**A**). Active mitochondria are shown by MitoTracker Red (Orange) and the total mitochondrial mass by MitoTracker (green). Digital Phase Contrast (Gray) has been added to show the cells shapes, also contoured in gray. Nuclei are stained in blue (Hoechst). Scale Bars = 10μm. Mitochondrial ratio (**B**), mass (**C**) and membrane potential (**D**). Data represented the mean of three independent experiments and results are shown as the mean ± SEM. Statistical significance: * *p* < 0.05, **p < 0.01 (One-way ANOVA test with Tukey’s correction). Representative Western blot analysis of OPA1 (80-100 kDa) and MFF (25–35 kDa) (**E**) and their expression levels in unrelated controls and TARDBP-ALS family fibroblasts compared to loading control (Vinculin 121 kDa) (**F**, **G**).

Since mitochondrial morphology affects function, we evaluated morphological and functional parameters of mitochondria such as the total mass and the ratio between active/total mitochondria. High-content analyses showed that controls and TDP-43^G376D^ carriers were indistinguishable based on these two metrics (Fig. 5A–C). However, a higher mitochondrial membrane potential, indicating a faster electron transport, was observed in *TARDBP* family fibroblasts, especially in patient cells (significantly different from unrelated CTRL) (Fig. 5D).

Given the fact that the balance between mitochondrial fusion and fission is vital for cellular homeostasis and that disturbances in these processes have increasingly been implicated in the pathogenesis of various neurodegenerative disorders [28], the expression level of OPA1, a mitochondrial fusion regulator, and MFF, involved in the mitochondrial fission complex, were investigated. All *TARDBP* family fibroblasts, apart from the late-stage patient fibroblasts, had a higher expression level of OPA1 than unrelated controls, while MFF content was slightly higher only in TDP-43^G376D^ Asymptomatic and late-stage patient fibroblasts compared to both unrelated and familial CTRLs (Fig. 5E–G and Supplemental Material).

### The dependency from metabolic substrates reflects the energetic flexibility of ALS fibroblasts alongside disease progression

**Fig. 6:**
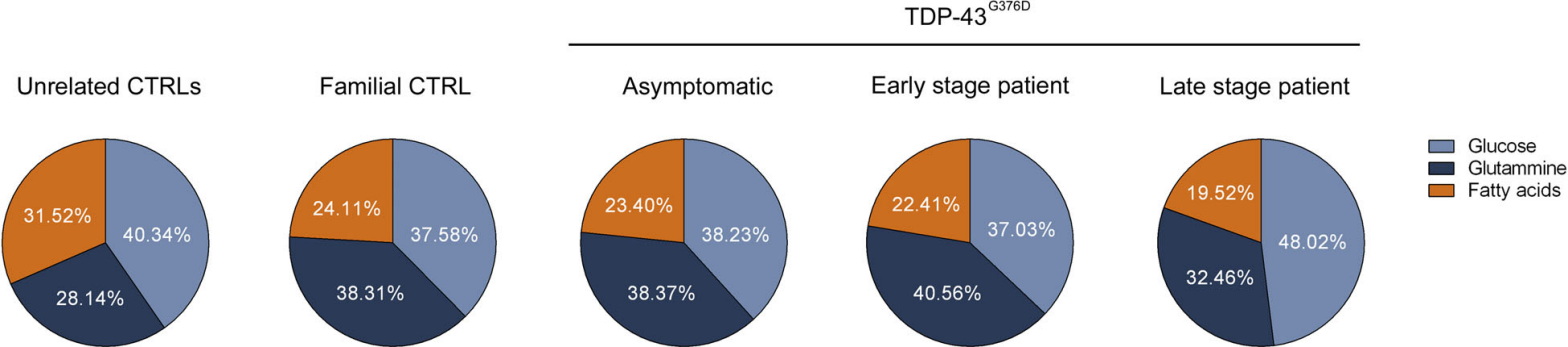
Evaluation of mitochondrial fuel oxidation in controls and TDP-43^G376D^ mutant fibroblasts. Pie charts indicate the relative dependency of cells on glucose, long-chain fatty acids and glutamine. Charts represent the results of three independent experiments. Following one-way ANOVA test with Tukey’s correction only fatty acids dependency showed a *p*-value of 0.0049 for Familial CTRL, 0.0024 for Asymptomatic fibroblasts, 0.0026 for early-stage fibroblasts and <0.0001 for the late-stage fibroblasts compared to unrelated CTRLs.

Adaptation to environmental changes and energetic demand involves several cellular mechanisms including changes in fuel choices [29]. We thus investigated the level of metabolic flexibility of the ALS fibroblasts by evaluating their dependence on glucose, long-chain fatty acids and glutamine, which are the main fuels oxidized by mitochondria (Fig. 6).

Unrelated control fibroblasts relied slightly more on glucose than glutamine or fatty acids for mitochondrial energy production. Interestingly, all *TARDBP* family fibroblasts showed an increased reliance on glutamine, suggesting an anaplerotic use of Krebs cycle. Moreover, they reduced their dependency from fatty acids compared to the unrelated controls (*p* = 0.0049 for Familial CTRL, *p* = 0.0024 for Asymptomatic fibroblasts, *p* = 0.0026 for early-stage fibroblasts *p* < 0.0001 for the late-stage fibroblasts). Of note, late-stage ALS fibroblasts showed a 10% higher reliance on glucose, in accordance with the hyper-glycolytic state of these cells.

## Discussion

In this study we have evaluated oxidative stress and energy metabolism of dermal fibroblasts derived from a fALS Italian family bearing the c.1127 G → A [p.G376D] mutation in the *TARDBP* gene. This mutation hits the glycine-rich domain at the C-terminus of TDP-43 and is associated with fALS cases with early onset and characterized by predominant upper limb impairment and aggressive course of the disease [16, 25, 30]. Within the family, the penetrance is high but below 100%, thus enabling the comparison between patients and asymptomatic TDP-43^G376D^ carriers. Indeed, cells from this unique familiar milieu have been leveraged to demonstrate that p.G376D causes cytoplasmic mislocalization of TDP-43 and pathological loss of the nuclear function of the protein [31] and to identify ALS progression biomarkers in blood cells [25]. We compared the bioenergetic profiles of primary fibroblasts from a TDP-43^G376D^ patient and healthy family members (both TDP-43^G376D^ carrier and non-carrier) with those of healthy unrelated controls. Our study uncovered the presence of common bioenergetic alterations shared within *TARDBP* family cells, and more specific features inherent to patient cells that mark the early-to-late disease stage transition.

We found that the cells form the *TARDBP* family have an impaired defense system against oxidative stress, with lower glutathione levels respect to the unrelated CTRLs, suggesting reduced abilities to cope with oxidative stress, which may account for the increase in ROS levels, despite the upregulation of SOD2 expression in patient’s cells. Low glutathione levels have been reported in various neurodegenerative diseases, including ALS [32, 33]. Reduced total glutathione levels may indicate a depletion of antioxidant defenses as a result of disease progression, or it may represent a predisposing factor that is exacerbated by genetic or environmental cues, thus contributing to disease pathogenesis. Our study suggests that this might be the case for the *TARDBP*-family, in fact low total glutathione is a feature of patient’s cells that is shared with family controls (both mutation carrier and non-carrier). The high ROS content could also be the consequence of mitochondrial dysfunctionality, leading to the upregulation of mitochondrial fusion, as suggested by higher OPA1 fusion protein levels in fibroblasts from *TARDBP* family members, a strategy often used to improve electron transfer rate [34, 35]. This increases both basal and maximal respiration rates observed in all the cells from *TARDBP* family members.

In addition to the elevated oxidative burden, the hyperproliferative phenotype and the augmented respiration rate, and in accordance with the systemic “hypermetabolism” theory of ALS patients [11], our data also show that *TARDBP* family fibroblasts display a higher total ATP production rate. Interestingly, this *TARDBP* family common trait is achieved by different metabolic rearrangements, peculiar for each cell line.

The *TARDBP* familial CTRL fibroblasts, unlike unrelated CTRLs cells, rely more on glycolysis than on oxidative phosphorylation for ATP production, despite the loss of efficiency, due to impaired electron transfer and mitochondrial ATP production that slow down the Krebs’ cycle. Moreover, respect to unrelated CTRLs, familial CTRL mitochondria depend more on glutamine as a fuel, than on glucose and fatty acids, a pattern which is found in all family members, except the late-stage patient. This normally implies that Krebs’ cycle is functioning in an anaplerotic way, with glutamine being converted into glutamate and glutamate yielding a-ketoglutarate, which enters the cycle in an anticlockwise fashion and is converted into citrate. The latter is exported into the cytosol to be carboxylated and acylated to yield malonyl CoA, which is used in fatty acid synthesis. An alternative hypothesis could be that alpha-ketoglutarate is used for substrate-level phosphorylation and then expelled from the cycle either through the malate–alpha-ketoglutarate transporter as malate or via the glutamate-aspartate shuttle as aspartate, leading to increased NADH level inside mitochondria. This could maximize ATP production from available substrates, fitting the observed hypermetabolic phenotype. Increased NADH availability could also explain lesser need to exploit fatty acids as mitochondrial fuel, in accordance with what observed in Mitofuel experiments. However, additional experiments, should be conducted in order to confirm the actual mechanisms underlying this phenotype. Fibroblasts from the asymptomatic TDP-43^G376D^ carrier show further metabolic alterations: mitochondrial respiration is enhanced, and the cells rely more on mitochondrial than on glycolytic ATP production; this is obtained by increasing both maximal and spare respiratory capacity, while basal respiration remains unchanged, suggesting that mitochondria are not working at the maximum level. The increase in ROS production, induced by oxidative phosphorylation upregulation, is matched by a further increase of SOD2 expression, respect to familial CTRL. However, no differences are observed in fuel preferences, respect to familial CTRL.

Different metabolic rearrangements are triggered in the ALS patient at the different disease stages. Our data show a further increase in fibroblasts proliferation sustained by a more energetic state, maintained throughout both the early and the late stage of the disease. These metabolic alterations alongside disease progression, lead to an increase of the patient metabolic potential, which becomes more energetic and more glycolytic, sustained by GAPDH upregulation. Nevertheless, at the early ALS stage, most of the ATP is produced by oxidative phosphorylation, which is still functioning, despite glycolysis upregulation. In fact, the Mito Stress Test shows a higher basal respiration rate, compared to TDP-43^G376D^ asymptomatic fibroblasts, but a much lower spare respiratory capacity, suggesting that the patient fibroblasts function at their maximum rate; the membrane potential is also accordingly increased in the patients’ fibroblasts. Moreover, while total ROS level remains unchanged, an increase in O_2_^-^ concentration is observed, respect to TDP-43^G376D^ asymptomatic fibroblasts, which is matched by a further increase in SOD enzymes. No significant differences are observed in mitochondrial fuel dependency, compared to familial control fibroblasts.

At the late stage of the disease another change in ATP production takes place with the majority of ATP being produced through glycolysis. Mitochondrial basal respiration is lower respect to the early stage, while both maximal respiration and spare respiratory capacity are higher, showing that mitochondria are working below maximum rate. In late-stage patient fibroblasts the increase in O_2_^-^ production is not balanced by SOD enzymes upregulation and this likely leads to more serious mitochondrial respiration impairment; in fact, both mitochondrial fusion and membrane potential increase seem to have reached their maximum level at the early stage of the disease. Consequently, mitochondria fail to deliver a sufficient amount of ATP, thus leading to an upregulating of glycolysis, as shown also by a new change in fuel dependency, with glucose becoming the main fuel and supporting hyperactivated glycolysis. Upregulation of glycolysis has been shown also in fibroblasts with mutations in SOD1 [33, 36], that switch from mitochondrial respiration to glycolysis to meet energy demands. Interestingly, alteration to metabolic processes controlling carbon flow into the mitochondria have been recently associated to TDP-43 mutations leading to the protein truncation [37]. In this context, our data show that dysfunctions of carbon fuels metabolism can be a pre-existing condition in fALS families that are managed by non-carriers or asymptomatic mutation carriers with mechanisms that cannot be sustained for extended time in ALS patient cells.

Although it is not possible at this stage to identify the mechanisms at the basis of the upregulation of mitochondrial respiration promoted by the presence of the TDP-43^G376D^, it is likely that the increased amount of ATP required by the cells to counteract the effect of the mutation drives the upregulation of mitochondrial respiration. Increased ATP could be obtained from glutamine conversion into glutamate, alpha-ketoglutarate and malate which is oxidized yielding additional NADH which fuels electron transport and ATP production; this is well in accordance with glutamine being the main mitochondrial fuel in patient fibroblasts at the early stage of the disease. However, aerobic respiration upregulation cannot last for a long time; it is effective in the TDP-43^G376D^ asymptomatic and in the early-stage patient but fails to match ATP requirement at a later stage, when ATP is mainly obtained from glucose in the glycolytic pathway.

Several reports [38, 39] point to a role of mutated TDP-43 in alterations of mitochondria functionality including ETC complexes impairments, mitophagy and morphometric alterations hence impacting on oxidative phosphorylation and overall energy metabolism. Despite a common consensus on metabolic defects, specific features have been described in ALS fibroblasts depending on TDP-43 mutations. Intriguingly, Allen et al. [40]. have described TCA cycle checkpoint alterations in the p.Y374X truncation determining glycolysis and mitochondrial compromission, depending on the metabolic intermediates used to feed the cells. Moreover, two studies performed on the p.A382T mutation [38, 39] have reported common bioenergetic features such as mitochondrial fragmentation, but also discrepancies in OCR values and mitochondrial ROS content. Considering this context and the paucity of comparable studies our data corroborate and give additional insights to the recent literature pointing to a key role of TDP-43 mutations in the compromission of cell bioenergetics. The variability of phenotypes observed between mutations and also within the same mutation, might reflect the importance of patient-specific studies, to uncover additional genetic/environmental contributing factors that can impact on the final disease phenotype. In light of these considerations, the peculiar cohort of donors described in this study enabled the detection of inherent family features indicating how this familial signature might represent the underlying predisposing background prodromic to the development of additional bioenergetic alterations displayed by ALS patient’s fibroblasts.

Although the use of fibroblasts allowed to understand the general metabolic alterations occurring in the TDP-43^G376D^ carrying cells, and particularly the role of oxidative stress, further studies on more complex cell models of ALS should be performed, including metabolomic analysis that will complement Seahorse data.

## Materials and Methods

### Collection of skin biopsies and primary skin fibroblasts cultures and growth curves

Primary fibroblasts have been obtained by dermal skin biopsies from healthy volunteers and from members of a *TARDBP* ALS-family non-carrier as well as p.G376D mutation carriers (both asymptomatic and symptomatic at different stages of the disease). All the enrolled donors signed an informed consent form for tissue procurement, previously approved by the Ethics Committee Palermo 1, 04/19 and Università Cattolica del Sacro Cuore A1320/CE/2012, Casa Sollievo della Sofferenza 136/CE before the collection of biological samples.

The biopsies have been collected by using a disposable biopsy punch of 4 to 5 mm in diameter and transferred in a sterile solution. To isolate fibroblasts, the skin fragments have been mechanically cut in smaller pieces and plated on a 35 mm tissue-culture dish with fetal bovine serum (FBS) at 37 °C, 5% CO_2_, overnight. The day after the fragments were transferred to Dulbecco’s Modified Eagle Medium (DMEM) with high glucose concentration supplemented with 20% FBS, 2 mM L-glutamine, 100 U/mL penicillin, 100 μg/mL streptomycin and 1% non-essential amino acids. They’ve been cultured under these normal primary fibroblasts’ conditions until their sprouting from the skin fragments. Cells have been cultured in a 100 mm tissue-culture dish and splitted when reached about 70% of confluency. Cellular proliferation was measured by plating 30 000 cells per well in a 12-multiwell plate and counted after 24, 48, 72 and 96 h.

All chemicals were supplied by Merck KGaA, Darmstadt, Germany.

Negativity for mycoplasma contamination was routinely assessed by mean of the N-Grade Mycoplasma PCR Kit (Euroclone, Pero, Milan, Italy) that provides reaction mixture of ingredients necessary for amplification, including the positive control.

### Energetic profile measurements

Cell energy phenotype and total ATP production were investigated using Agilent Seahorse XFe96 Analyzer according to manufacturer protocols. Cell seeding densities were optimized according to Seahorse XF Analyzer recommendation protocol, based on OCR measurements from calibration experiments.

Fibroblasts derived from unrelated controls were seeded at a density of 2 × 10^4^ cells per well, while cells from familial control, asymptomatic mutation carriers and early and late-stage mutant patient were seeded at a density of 4 × 10^4^ cells per well in Seahorse XF Cell Culture Microplates. Cell seeding was performed in 180 μL of growth medium and cells were allowed to adhere for 1 h at room temperature, before incubating them in a 37 °C humidified incubator with 5% CO_2_ for 24 h. In addition, the Seahorse XF Sensor Cartridge was hydrated with sterile water the day before running the assay and calibrated with 200 μL per well of Seahorse XF Calibrant Solution the day of the experiment in a non-CO_2_ 37 °C incubator. Cell energy profile and ATP production were investigated by using Agilent Seahorse XF Cell Energy Phenotype Test Kit and Agilent Seahorse XF ATP Rate Test Kit, respectively.

Normalization was performed on total protein content, measured with Bradford assay [41].

All the kits and reagents were purchased by Agilent Technologies (Santa Clara, CA, USA).

### Western blot analysis

Pellets of 1 × 10^6^ derived from fibroblasts were collected. Cellular pellets were lysed with RIPA buffer (Tris 50 mM, NaCl 150 mM, NP40 1%, SDS 0.1%, sodium deoxycholate 0.5%, pH 7.5), containing protease inhibitors (1 μM leupeptin, 2 μg/mL aprotinin, 1 μg/mL pepstatin and 1 mM PMSF) and incubated on ice for 15 min. Then homogenates were obtained by passing 5 times through a blunt 20-gauge needle fitted to a syringe and centrifuged at 13,000 rpm for 20 min at 4 °C. Subsequently, supernatants were collected, and the total protein amount was quantified using the bicinchoninic acid (BCA) protein assay kit according to the manufacturer’s protocol.

SDS-PAGE and Western blot were performed by standard procedures. 35 μg of proteins were loaded on the separating 10 or 12% acrylamide/bis-acrylamide SDS-PAGE and transferred onto a nitrocellulose membrane (Millipore, Billerica, MA, USA), which was then blocked for 30 min in a 5% non-fat milk powder in a PBS solution and incubated overnight at 4 °C with the primary antibody. The following primary antibodies had been used: anti-SOD1 (dilution 1:1000, NBP1-47443, Novus Biologicals, Centennial, CO, USA), anti-SOD2 (dilution 1:1000, #13141 Cell Signaling Technology, Danvers, MA, USA), anti-GAPDH (dilution 1:1000, #2118 Cell Signaling Technology, Danvers, MA, USA), anti-OPA1 (dilution 1:1000, #80471 Cell Signaling Technology, Danvers, MA, USA), anti-MFF (dilution 1:1000, #84580 Cell Signaling Technology, Danvers, MA, USA) and anti-vinculin (dilution 1:5000, V9131 Merck KGaA, Darmstadt, Germany). Nitrocellulose membranes were washed three times for 10 min with TBS with 0.3% (v/v) Tween 20, incubated for 1 h with anti-mouse IgG HRP-conjugated secondary antibodies (#7076 Cell Signaling Technology, Danvers, MA, USA) diluted 1:8000 or anti-rabbit IgG HRP-conjugated secondary antibodies (#7074 Cell Signaling Technology, Danvers, MA, USA) diluted 1:5000. After additional three 10 min washings in TBS with 0.3% (v/v) Tween 20, protein signals were revealed using the Chemiluminescence (ECL) detection system (EuroClone, Pero, Milan, Italy). Finally, protein levels were quantified by densitometry of optical density of the bands using ImageJ software (National Institute of Health, MD, USA) and normalized on loading control.

### Mitochondrial functionality and fuel dependency analysis

Fibroblasts were firstly seeded in Seahorse XF Cell Culture Microplates at a density of 2 × 10^4^ cells per well for unrelated controls and 4 × 10^4^ cells per well for familial control and mutation carriers (both asymptomatic and symptomatic) in 180 μL of growth medium, then allowed to adhere for 1 h at room temperature and finally incubated for 24 h in a 37 °C humidified incubator with 5% CO_2_. In addition, the day before running the assay, the Seahorse XF Sensor Cartridge was hydrated with sterile water followed by the calibration step (200 μL per well of Seahorse XF Calibrant Solution) the day of the experiment in a non-CO_2_ 37 °C incubator.

Mitochondrial basal and maximum respiration as well as coupling efficiency were evaluated with Agilent Seahorse XF Cell Mito Stress Test Kit, while mitochondrial fuel dependency analysis has been carried out with Agilent Seahorse XF Mito Fuel Flex Test Kit.

Normalization was performed on total protein content, measured with Bradford assay [41].

All the kits and reagents were purchased by Agilent Technologies (Santa Clara, CA, USA).

### Glutathione and Intracellular Reactive Oxygen Species (ROS) Detection

Total intracellular glutathione (GSH) has been measured as described in Bovio et al., 2021[42], by using 50 μL of cell lysate for each fibroblasts cell line.

Total intracellular reactive oxygen species (ROS) were detected with dichlorofluorescin diacetate (H_2_DCFDA) probe, while cytosolic superoxide anion (O_2_^-^) content was measured using the fluorogenic probe dihydroethidium (DHE).

For these experiments, fibroblasts were seeded in 96-well black microplates with clear bottom at a density of 1 × 10^4^ cells per well and cultured in complete DMEM medium. 24 h after the seeding, they were incubated with 5 μM H_2_DCFDA or 10 μM DHE in PBS (10 mM K_2_HPO_4_, 150 mM NaCl, pH 7.2) for 30 min at 37 °C, 5% CO_2_ in the dark. Plates were then rinsed in PBS twice and fluorescence was measured, in end point mode, using a fluorescence microtiter plate reader (VICTOR X3, PerkinElmer, Akron, OH, USA) at emission 485 nm/excitation 535 nm for H_2_DCFDA and at emission 490 nm/excitation 590 nm for DHE.

Normalization was performed on total protein content, measured with Bradford assay [41].

All chemicals were supplied by Merck KGaA, Darmstadt, Germany.

### Live cell mitochondrial fluorescence measurements using high-content microscopy

We exploited the use of Operetta high-content analysis to measure the total mitochondria mass and the percentage of active mitochondria over the total. To the scope we used a combination of MitotrackerTMDyes for Mitochondria Labeling series by Thermofisher that has been specifically designed for multicolor labeling experiments [43–45]. More specifically we used: MitoTracker Green FM (M7514, Thermo Fisher Scientific, MA, USA), which stains the total mitochondria mass and MitoTracker Red CMXRos (M7512, Thermo Fisher Scientific, MA, USA) that stains mitochondria in live cells and accumulates selectively in mitochondria due to its negative membrane potential. These dyes link to thiol groups in the mitochondria, thus can be used in experiments in which multiple labeling might diminish mitochondrial function. Fibroblasts were seeded in a 96-well imaging plates (CELLSTAR black µCLEAR, Greiner Bio-One) at a density of 3 × 10^3^ cells per well and cultured for 24 h at 37 °C in a 5% CO_2_ incubator in complete high glucose DMEM supplemented with 20% FBS. Culture media were then removed and replaced with fresh growth medium with a fluorophore dye cocktail containing 100 nM MitoTracker Green FM, 50 nM MitoTracker Red CMXRos and 1 μg/mL Hoechst 33342 (B2261, Sigma Aldrich, St. Louis, MO, USA). After 30 min of incubation at 37 °C in a 5% CO_2_ incubator, cells were washed twice with HEPES-buffered salt solution (HBSS) prior to starting live-cell imaging at Operetta CLS™ high-content analysis system (PerkinElmer, Waltham, MA, USA). Images were analyzed using Harmony 4.9 software.

Image acquisition was performed with a 40 × water-immersion objective. For each subject, fifty-four Fields of View (FoV) composed by maximum projection of eight z-stack images, were randomly taken and analyzed using Harmony software, as described below.

For each Fov, digital phase contrast (DPC) was used to detect fibroblasts and calculate the total area covered by the cells (by “Find Cell” building block); MitoTracker Green signal was used to identify and calculate the total area covered by mitochondria (by using ‘Filter and Find Image’ and ‘calculate morphology and properties’ building blocks). The ratio between the total area covered by mitochondria and the total area covered by cells was taken as an estimate of the mitochondrial mass.

For each FoV, MitoTracker Red CMXRos was used to detect and calculate the total active mitochondria area (by using the ‘Filter and Find Image’ and ‘calculate morphology and properties’ building blocks). The ratio between the total active mitochondria area over the total mitochondria area, was used as a measure of the active fraction of mitochondria.

### Mitochondrial Transmembrane Potential (MTP) assay

MTP was assessed using the mitochondrial potential sensitive carbocyanine dye 3,3′-dihexyloxacarbocyanine iodide (DiOC6) [46, 47], which accumulates in mitochondria due to their negative membrane potential. The cells were plated in complete DMEM medium at a density of 1 × 10^4^ cells per well in 96-well black microplates with clear bottom and after 24 h incubated with 40 nM DiOC6 in PBS for 20 min at 37 °C and 5% CO_2_ in the dark. Plates were rinsed in PBS twice and fluorescence was measured at emission 485 nm/excitation 535 nm in end point mode, using a fluorescence microtiter plate reader (VICTOR X3, PerkinElmer, Akron, OH, USA).

Normalization was performed on total protein content, measured with Bradford assay [41].

All chemicals were supplied by Merck KGaA, Darmstadt, Germany.

### Statistical analysis

Data analysis has been carried out with GraphPad Prism software (GraphPad Software Version 8, Inc., San Diego, CA, USA). Data were presented as the mean squared error (SEM). One-way ANOVA test with Tukey’s correction was used to evaluate statistical significance within the different samples. For statistical analyses, for each cell line at least three biological replicates were measured for each independent experiment. The number of independent experiments for each analysis is described in the figure caption. Results were considered statistically significant at *p* < 0.05.

## Supplementary information

Original Data

## Data Availability

All data generated or analysed during this study are included in this published article. Data analysed will be made available from the corresponding authors on reasonable request, unless for the confidential medical records and related.
